# Burnout syndrome prevalence during internship in public and private hospitals: a survey study in Mexico

**DOI:** 10.1080/10872981.2019.1593785

**Published:** 2019-04-08

**Authors:** Roberto Carlos Miranda-Ackerman, Francisco José Barbosa-Camacho, María José Sander-Möller, Arturo David Buenrostro-Jiménez, Roberto Mares-País, Ana Olivia Cortes-Flores, Gilberto Morgan-Villela, Carlos José Zuloaga-Fernández del Valle, Manuel Solano-Genesta, Clotilde Fuentes-Orozco, Guillermo Alonso Cervantes-Cardona, Gabino Cervantes-Guevara, Alejandro González-Ojeda

**Affiliations:** a Hospital San Javier, Unidad de Cuidados Intensivos, Guadalajara, Jalisco, México; b Departamento de Educación e Investigación, Hospital de Especialidades del Centro Médico Puerta de Hierro Norte, Zapopan, Jalisco, México; c Unidad de Investigación Biomédica 02 Hospital de Especialidades, Centro Médico Nacional de Occidente, Instituto Mexicano del Seguro Social, Guadalajara, Jalisco, México; d Centro Universitario de Ciencias de la Salud, Universidad de Guadalajara, Guadalajara, Jalisco, México; e Centro Universitario del Norte, Universidad de Guadalajara, Colotlán, Jalisco, México

**Keywords:** Burnout syndrome, health professional, internship, mental health, medical student

## Abstract

Burnout syndrome is a psychological condition that commonly affects health professionals, medical students, and others in professions with long shifts. It is defined by a high amount of emotional exhaustion, depersonalization, and low personal job satisfaction. We aimed to determine the prevalence of burnout syndrome in medical interns and establish the relationships between this condition and the time and type of hospital at which students worked during their medical internship. This was a survey study in which we used the Maslach Burnout Inventory, applied to fifth-year medical students on an internship at private and public hospitals in Mexico. The participants were 96 women (54.5%) and 80 men (45.5%), with ages ranging from 21 to 34 years old. We found burnout syndrome in 20% of these medical students 22% of the women and 18.6% of the men in the sample. Second-semester interns suffered burnout at a rate of 29%, in contrast to 15% of first-semester students. Emotional exhaustion and depersonalization scores were higher in second-semester interns who worked in public hospitals. However, the prevalence did not differ between public and private hospitals. Our study reports a higher prevalence of burnout syndrome during the second semester of internship. Students who practiced their internship in a public hospital showed higher scores in emotional exhaustion and depersonalization than those who practiced in a private hospital.

## Introduction

Burnout syndrome is a psychological condition characterized by emotional exhaustion, depersonalization, and low personal accomplishment, which translates to inefficient job performance. It has been associated with high-stress jobs, jobs that involve taking care of people, and jobs with long working hours; by definition, it is a common problem among health professionals and caretakers [1]. Burnout syndrome is studied and identified by three different dimensions: emotional exhaustion, which measures feelings of being emotionally overextended and exhausted by the work; depersonalization, which measures an unfeeling and impersonal response towards the person receiving treatment or service; and personal accomplishment, which measures feelings of competence and achievement in the person’s work [2].

In Mexico, it is mandatory that every medical student has a one-year internship at a public or private hospital within the Health Care Network. This is done to reinforce and apply the medical knowledge that students have gained during medical school [3]. This internship must be completed before applying for the professional exam and a year of social service. During this time, the medical student works as a key member of personnel at a hospital of their choice, with options across the country, including both public- and private-sector facilities.

Starting with those with the highest grade-point average, students are given the opportunity to select which hospital to practice their internship in. Students choose from a list of hospitals within the affiliations of each medical school across the country. Although most of the hospitals available are public hospitals, such as those within the Mexican Institute of Social Security and the Institute of Security and Social Services of the State Workforce networks, some medical schools have affiliations with private hospitals to provide medical students with the opportunity to learn about private practice.

To medical students, this period of time is extremely stressful because aside from studying for tests and evaluations, they must complete training in different areas, such as surgery, gynecology, family medicine, internal medicine, pediatrics, and emergency rooms. They start to take on patient responsibilities, including attending hospitalized and ambulatory patients, assisting with or performing surgeries, taking blood samples, and writing notes for the medical record. These responsibilities also include being on call for 24–36 hours, and during this on-call time, students face stressful and demanding situations, irregular eating schedules, sleep deprivation, and – in some cases – humiliation or gender discrimination by attending doctors or nursing staff, either for personal reasons or to reinforce a hierarchy [4,5].

Although internships should be similar across hospitals, there are differences between private and public hospitals. Everything from administration aspects to patient characteristics between hospitals influences the skills their interns develop.

Public hospitals are more demanding workplaces because their patient populations are far more extensive than those of private hospitals; hence, interns at these hospitals are needed more extensively for medical consultation and in emergency and operation rooms (along with the attending medical staff), thus exposing them to greater stress and sleep deprivation. In addition, because of a lack of hospital staff (ranging from nurses to patient transport personnel), medical students may have to take on extra responsibilities to replace or support other roles to guarantee patient care. This means medical students have even less time to sleep, and may be unable to attend educational sessions or, in some cases, eat their meals. This contrasts with private hospitals, where interns are more likely to further develop their academic abilities than their bedside clinical skills. This difference may be a result of having a smaller patient population and sufficient numbers of medical and nonmedical staff.

In countries such as the USA, regulations have created conditions in which medical residents are supported, for example, by restricting working hours [6]. These regulations have also facilitated care for the mental and physical health of medical students, interns, and residents, in part to help reduce medical errors and iatrogenic decisions due to sleep deprivation and mental fatigue. Similarly, as of November 2018, the Mexican Government Secretariat passed a law to change medical interns’ hospital shift schedules (NOM-001-SSA3-2018) [7].

Interns and residents used to be on-call once every three days, sometimes having three calls a week. Now, thanks to the new law, interns and residents are on-call one out of every four days, have a maximum of two calls each week, and a limit of 80 hours of work per week. In theory, this should reduce demands on medical students and decrease their levels of stress and burnout.

## Materials and methods

### Aims

This study aimed to determine the prevalence of medical intern burnout syndrome, and whether a relationship exists between this condition and the duration of internship or type of hospital where interns work. Understanding this relationship may facilitate the development or improvement of strategies to reduce the prevalence of burnout syndrome and to improve medical students’ quality of life.

### Study design

This was a cross-sectional survey study that evaluated the presence of burnout syndrome using the Maslach Burnout Inventory (MBI) (S1 Appendix) [8], in samples of medical students practicing their internship at private and public hospitals.

The sample size was based on burnout syndrome prevalence, in accordance with the protocol published by Athié-Gutiérrez and colleagues [9]. A minimum sample size of 144 individuals was calculated using an α error of 0.05 and β of 0.20. The sample included 176 medical students who were practicing their one-year internship at two public and two private hospitals. We made physical and online forms of the survey and distributed it to the medical interns working in the authors’ hospitals. All the students answered the survey once during their internship. We included 68 students from two different private hospitals (which represented 100% of their total interns), and 108 students from two different public hospitals (which represented 70% of the public hospitals’ total interns).

### Instrument

We used the Maslach Burnout Inventory (MBI) [8], a 22-item questionnaire that includes evaluation of three subscores:

- Emotional exhaustion, which includes nine items with a maximum of 54 points (items 1, 3, 6, 8, 13, 14, 16, and 20)
- Depersonalization, which includes five items with a maximum of 30 points (items 5, 10, 11, 15, and 22)
- Personal accomplishment, which includes eight items with a maximum of 48 points (items 4, 7, 9, 12, 17–19, and 21).

Burnout syndrome requires high scores on emotional exhaustion (> 26) and depersonalization (> 9), and a low score on personal accomplishment (< 34) for an accurate diagnosis. We analyzed the prevalence of burnout syndrome as well as each of the syndrome’s subscores.

The survey also inquired about the students’ gender, age, whether they were undertaking practice in their first semester (first six months of internship) or second semester (last six months of internship), and the type of hospital they were attending.

### Data analysis

Data were analyzed using the Statistical Package for Social Sciences (IBM Corp. Armonk, NY), Version 23.0 for Windows. Descriptive analyses included proportions, means, and standard deviations. Inferential analysis was performed using the chi-square test or Fisher’s exact probability test or ANOVA test, as appropriate for categorical variables. Student’s t-test was used to analyze continuous variables. A probability level of *p* < 0.05 was considered statistically significant.

### Ethical considerations

Written consent was obtained from each research participant before the study, ensuring the protection of their privacy, confidentiality, and anonymity. The study protocol was approved by the Ethics and Research Committee of the Hospital de Especialidades of the Centro Médico Puerta de Hierro with the register HECMPDH/2018/PROT/001. The authors assert that all procedures contributing to this work comply with the ethical standards of the relevant national and institutional committees on human experimentation in accordance with the Helsinki Declaration and the Mexican Health Guidelines.

## Results

The participants were 176 Mexican medical students, 96 women (54.5%) and 80 men (45.5%). Their mean age was 23.07 ± 1.4 years (range 21–34 years), and their median age was 23 years. Approximately one-third (34.2%) of the participants were working at a private hospital, and two-thirds (65.7%) were working at a public hospital. Burnout syndrome prevalence was 20% (n = 35), with 21 women (21.8%) and 14 men (17.5%). Most of these students were younger than 25 years (n = 161, 91.5%) with only 15 (8.5%) aged 25 years or older. Burnout syndrome was present among 33 of the younger students, and two of the older students; however, this was not a statistically significant difference (*p* = 0.738). Seventeen (15%) of the students affected by burnout syndrome were in their first semester of the internship; 18 (29%) were in their second semester (*p* = 0.02), as shown in Table 1.

**Table 1.** Distribution of Burnout syndrome among fifth-year medical students.

	Students with burnout syndrome *n *= 35 (20%)	Students without burnout syndrome *n *= 141 (80%)	*p*-value
Male Female	14 (18.6%) 21 (22%)	61 (81.4%) 75 (78%)	0.57
First semester Second semester	17 (15%) 18 (29%)	97 (85%) 44 (71%)	0.02*
Private hospital Public hospital	12 (17.6%) 23 (21.3%)	56 (82.4%) 85 (78.7%)	0.55

* *p* ≤ 0.05 was considered statistically significant.

Analysis of the emotional exhaustion subscores showed that 76 students (43.1%) presented high scores on emotional exhaustion. Forty-three (56.5%) of the medical students with high scores on emotional exhaustion were women, and 33 (43.4%) were men, which was not a statistically significant difference (*p* = 0.650). Of these 76 students, 42 (55.2%) students were in their first semester of internship, while 34 (44.7%) were in their second semester. We observed the highest scores in second-semester students (*p* = 0.002) and those working in public hospitals (*p* = 0.04).

On the depersonalization subscore, 95 students (53.9%) presented high scores. Forty-eight (50.5%) of the medical students with high depersonalization subscores were women, and 47 (49.4%) were men, which was not a statistically significant difference (*p* = 0.288). Of these 95 students, 55 (57.8%) were in their first semester and 40 (42.1%) were in their second semester. We observed higher depersonalization scores in second-semester students (*p* = 0.009) and in those working in public hospitals (*p* = 0.009).

Sixty-one students (34.6%) presented a low personal accomplishment score. Thirty of these medical students (49.1%) were women and 31 (50.8%) were men (not statistically significant, *p* = 0.341). Of these students, 38 (62.2%) were in their first semester and 23 (37.7%) were in their second semester (not statistically significant, *p* = 0.08). There was no statistically significant difference based on the type of hospital (*p* = 0.26), as shown in Table 2.

**Table 2.** Maslach Burnout inventory subscores according to gender, age, semester, and hospital type.

	Emotional exhaustion	*p*-value	Depersonalization	*p*-value	Personal accomplishment	*p*-value	Degree of freedom
Male	23.14 ± 9.69	0.274	9.96 ± 6.18	0.438	35.94 ± 6.99	0.279	174
Female	25.01 ± 12.45		9.23 ± 6.30		37.16 ± 7.88		
First semester	22.26 ± 11.24	0.002**	8.68 ± 6.25	0.009**	37.33 ± 7.24	0.087	174
Second semester	27.65 ± 10.60		11.19 ± 5.93		35.26 ± 7.82		
Private hospital	21.94 ± 11.58	0.041	8.01 ± 5.86	0.009**	37.40 ± 7.46	0.265	174
Public hospital	25.56 ± 10.92		10.54 ± 6.29		36.10 ± 7.51		

* *p* ≤ 0.05, ** *p* ≤ 0.0010. Both were considered statistically significant.

Fifty-one students (29%) did not present altered scores on any of the MBI subscores. The number of students with high scores on any subscore surpassed the number who had diagnostic-level burnout syndrome; this was because presenting one or two altered scores did not fulfill the diagnostic criteria.

## Discussion

Even though they are still medical students, interns may suffer from burnout syndrome. Our study suggests that this prevalence increases with the additional requirements of academic training and work time when serving a continually growing patient population.

Our findings suggest that the prevalence of this syndrome is relevant since, for every three students, one suffers burnout syndrome. As expected, this prevalence was more evident in interns practicing in public hospitals, where the workload is high, and little time is spent on education, rather than in interns in private hospitals. This is also reflected in the scores of MBI subscales, which are higher overall in emotional exhaustion and depersonalization.

We hypothesized that second-semester students would have a higher prevalence of burnout syndrome because of the length of time invested in the internship, reducing their tolerance towards patients and paperwork, and resulting in a decrement in patient care. While completing the survey, one intern said: ‘*The more time passes, the less I feel like my work matters around the hospital, and the less I feel engaged in the patients’ care’*.

Overall, second-semester interns showed higher scores in MBI subscores of emotional exhaustion and depersonalization and lower scores in personal accomplishment than first-semester interns.

The studied population was located in western Mexico, which is entirely different to the southern part of the county where there is a prominently marginalized population that lacks equipped healthcare centers, health professional staff, and supplies for all the citizens of towns and villages. This increases the necessity for medical interns in insecure and isolated parts of the country, where the stress experienced by students is even higher.

On MBI subscores, interns working at public hospitals showed higher scores on emotional exhaustion and depersonalization. Our overall sample had higher scores on depersonalization than several previous studies [9–11].

Our study showed a similar prevalence of burnout syndrome to studies from Mexico City and the UK, but much lower rates than in a US study [9–11]. A systematic review by Ishak et al. compared nine studies of students with burnout, finding a prevalence ranging between 45% and 71%. MBI subscores across these studies also had different distributions, as shown in Table 3.

**Table 3.** Prevalence of Burnout across previously published studies.

						Maslach Burnout Inventory		
						High scores		Low scores
	Mean age	Number of students	Years of training	Hospital setting	Burnout prevalence	Emotional exhaustion	Depersonalization	Personal accomplishment
Present study	23	176	4	Private/Public Settings	20%	43.10%	53.90%	34.60%
Mexico City [9]	23	141	4	Public Settings	16.30%	63.30%	40.40%	30.40%
UK [10]	23	47	4	Private Settings	26.70%	54.80%	34%	46.60%
USA [11]	25	887	4	Private/Public Settings	55.90%	44.60%	37.90%	35.80%

Ishak et al.’s review found emotional exhaustion ranged from 43.1% to 63.3%; our sample fell in the lower range. For depersonalization, it is interesting to note that our overall sample (i.e. with or without burnout) had an elevated subscore of 53.9%, exceeding the highest in the range of reviewed studies (34–40.4%). Our sample’s distribution of scores on personal accomplishment was similar to previous studies (30.40–46.60%) [9–12].

There are essential differences between medical students and other college students. The main difference is the requirement to be on call, which has been described as a primary factor increasing the potential for burnout [12]. Further evidence for this is provided by comparing results from studies of medical students with those of students following nonmedical careers (such as administration or psychology), among whom burnout syndrome prevalence ranges from 0 to 1.3% [13].

The transition from college to the workforce is a significant change in students’ lives, representing more significant responsibilities, a competitive job market, and an overall change in lifestyle. Further, most graduates have overly high or imprecise expectations about how they should approach work, resulting in an increased buildup of stress [14].

Because this is a frequent condition among medical students and healthcare professionals, prevention measures should be implemented within healthcare centers to decrease syndrome prevalence. As previously suggested by West et al., institutional and individualized interventions for personnel could help reduce the high incidence of burnout syndrome [15]. Moore has described a few strategies used by mental health professionals to avoid stress buildup and burnout, including leaving documentation at work, disconnecting from social interaction with coworkers, spending more time with friends and family, and taking time for self-care [16].

We believe burnout syndrome is an important issue to attend to as it affects our fellow medical students during their training to become doctors, and can abate the goodwill and desire to help patients as well as their enthusiasm for studying further. Dyrbye et al. indicate that burnout can influence thoughts of dropping out of medical school, especially in the third and fourth years [17].

It has been reported that burnout can influence residents and medical students to develop unprofessional behavior, such as omitting or neglecting information, and providing lower quality care [18]. In addition, an altered emotional state, such as burnout syndrome, can mitigate cognitive capabilities such as memory, interpretation of information, and skill acquisition [19].

Our population of medical students demonstrates that regardless of whether the hospital is public or private, burnout can affect their mental and emotional state. As mentioned before, this disorder could lead to faulty training and weak medical performance in the long term. We encourage further studies to identify the factors that lead to burnout across our current and future physicians.

## Copyright statement

Rights to use the Maslach Burnout Inventory were obtained by purchasing the license provided by Mind Garden®.
