# Supplementary material for: Burnout syndrome prevalence during internship in public and private hospitals: a survey study in Mexico
Source: Med Educ Online. 2019 Apr 8;24(1):1593785. doi: 10.1080/10872981.2019.1593785 (PMC6461102; doi:10.1080/10872981.2019.1593785)
Supplement: Supplemental Material [file ZMEO_A_1593785_SM6291.docx]

**S1 Appendix. The Maslach Burnout Inventory Survey*.**

Gender: ___________________________________________________________

First or second semester of internship: ___________________________________

Age: ______________________________________________________________

Private or public hospital: _____________________________________________

Indicate which answer you think more accurately describes the frequency for each sentence.

0=Never; 1=A few times a year; 2=A few times a month or less; 3=Sometimes a month; 4=Once a week; 5=A few times a week; 6=Every day

| 1 | I feel emotionally drained from my work. |  |
| --- | --- | --- |
| 2 | I feel used up at the end of the workday. |  |
| 3 | I feel fatigued when I get up in the morning and have to face another day on the job |  |
| 4 | I can easily understand how my patients feel about things. |  |
| 5 | I feel I treat some patients as if they were impersonal objects. |  |
| 6 | Working with people all day is really a strain for me. |  |
| 7 | I deal very effectively with the problems of my patients. |  |
| 8 | I feel burned out from my work. |  |
| 9 | I feel I'm positively influencing other people's lives through my work |  |
| 10 | I've become more callous toward people since I took this job. |  |
| 11 | I worry that this work is hardening me emotionally |  |
| 12 | I feel very energetic. |  |
| 13 | I feel frustrated by my work |  |
| 14 | I feel I'm working too hard on my job. |  |
| 15 | I don't really care what happens to some patients. |  |
| 16 | Working with people directly puts too much stress on me. |  |
| 17 | I can easily create a relaxed atmosphere with my patients. |  |
| 18 | I feel exhilarated after working closely with my patients. |  |
| 19 | I have accomplished many worthwhile things in this job. |  |
| 20 | I feel like I'm at the end of my rope. |  |
| 21 | In my work, I deal with emotional problems very calmly. |  |
| 22 | I feel patients blame me for some of their problems. |  |

***Survey under license of Mind Garden, Inc.**
